# The Association Between Dry Eye Disease With Depression, Anxiety and Sleep Disturbance During COVID-19

**DOI:** 10.3389/fpsyt.2021.802302

**Published:** 2022-01-05

**Authors:** Qing He, Zhuo Chen, Caiyuan Xie, Lin Liu, Ruihua Wei

**Affiliations:** Tianjin Key Laboratory of Retinal Functions and Diseases, Tianjin Branch of National Clinical Research Center for Ocular Disease, Eye Institute and School of Optometry, Tianjin Medical University Eye Hospital, Tianjin, China

**Keywords:** dry eye disease, anxiety, depression, dry, sleep, COVID-19

## Abstract

**Objective:** This study aimed to investigate the relationship between dry eye disease (DED) with anxiety and depression. Additionally, the mediating effect of sleep quality on this relationship was explored.

**Methods:** 321 patients with DED were recruited from Tianjin Medical University Eye Hospital clinic and surveyed using demographic questionnaires, the Ocular Surface Disease Index (OSDI), Hospital Anxiety and Depression Scale (HADS), and Pittsburgh Sleep Quality Index (PSQI). Regression analysis and the bootstrap method were used to investigate the influence of sleep on the relationship between DED, anxiety and depression.

**Results:** Among the patients with DED, 86 (26.79%), 85 (26.48%), and 54 (16.82%) patients presented with anxiety, depression, and both anxiety and depression respectively. The OSDI and PSQI score were positively correlated with depression and anxiety (all *p* < 0.01). The direct effects of OSDI on depression and anxiety were significant (*P* < 0.01). Additionally, the bootstrap test showed significant mediating effects of subjective sleep quality [95% CI [0.003–0.016] (depression); [0.001–0.011] (anxiety)] and sleep latency [95% CI [0.001–0.010] (depression); [0.001–0.008] (anxiety)]. These results indicated that the severity of DED symptoms, as measured by the OSDI score, affected anxiety and depression through a direct and an indirect pathway mediated by subjective sleep quality and sleep latency.

**Conclusions:** The results indicated that there was a significant correlation between DED and anxiety and depression. Moreover, subjective sleep quality and sleep latency were a mediator of the relationship between DED symptoms and anxiety and depression.

## Introduction

Since December 2019, coronavirus disease 2019 (COVID-19) has swept the world and has been declared a global public health emergency by the World Health Organization. COVID-19 is an infectious disease caused by severe acute respiratory syndrome corona virus 2 (SARS-COV-2) with droplets and contact as the main modes of transmission (1).COVID-19 is characterized by high infectivity and mortality. The cumulative number of confirmed cases worldwide was 200,174,883 and of deaths were 4,255,892, respectively, until August 5, 2021.

The widespread and rapid dissemination of COVID-19 has caused serious mental health stress to the public, such as anxiety, depression, and sleep problems (2–4). Meanwhile, during COVID-19 outbreak, individuals' isolation at home and the use of electronic devices have increased greatly (5).Long-term use of electronic products will inevitably cause eye diseases, such as dry eyes disease, myopia (6, 7).

Previous research (8) found ocular problems in COVID-19 patients, with conjunctival congestion as the primary symptom (9), while eye pain, photophobia and dry eye were also reported (10). Dry eye disease (DED) is a common ocular surface disease, with patients complaining of discomfort including dryness, foreign body sensation, burning sensation, photophobia, and eye pain, which can affect their the quality of life (11, 12). DED patients with chronic eye discomfort may also experience emotional problems such as anxiety and depression (13–15).

Several studies (16–19)have found the prevalence of depression in dry eye patients to be around 25–53.7% and anxiety around 39–63.6%. The severity of signs and symptoms in patients with DED did not change concomitantly. Kim et al. (20) found that the discomfort of DED and anxiety-depression were correlated; however, several studies concluded the opposite (21–23). Since the relationship between the symptoms of DED and anxiety and depression is unclear, it is crucial to conduct further research. Moreover, sleep disorders are another serious problem in patients with DED. In addition, patients with DED are more likely to have sleep problems than other eye diseases (18). Wu et al. (24) have found that DED patients with sleep disorders are more likely to experience anxiety and depression. Ayaki (25) found that subjective sleep quality was closely associated with anxiety and depression in women with DED. Sleep and mental problems in patients with DED have been the main interest of many researchers, but the precise association between the different components of sleep and anxiety and depression is not clear.

Various studies have found an increase in anxiety, depression, and sleep disorders during the COVID-19 epidemic (4, 26, 27). However, the relationship between sleep and mood problems in individuals with DED during the COVID-19 pandemic is unclear. Therefore, we want to investigate whether there is a correlation between DED, sleep, anxiety and depression. Furthermore, we try to explore the mediating effect of sleep on the relationship between DED and anxiety and depression in patients during the COVID-19 pandemic.

## Materials and Methods

The 321 patients with DED were recruited between May and July 2021 at Tianjin Medical University Eye Hospital in China. Approval was obtained from the Medical ETHICS Committee of Tianjin Medical University Eye Hospital. The procedures used in this study adhered to the tenets of the declaration of helsinki. informed consent was obtained from all participants included in the study.

Participants were literate adults aged >18 years with an Ocular Surface Disease Index (OSDI) score of ≥13 points. We excluded patients who had complications from anterior ocular segment diseases except for DED, patients with a history of ophthalmic surgery in the last 3 months, patients with severe glaucoma, bilateral cataract, and exclude systemic diseases such as autoimmune diseases, severe cardiopulmonary diseases, allergic diseases, and neurologic or psychiatric disorders. Previously diagnosed anxiety and depression were also excluded. In addition, patients who received anti-allergy drugs and contraceptives were excluded. Patients with an alcohol and drug dependence history, those taking anti-anxiety and antidepressant drugs, with serious medical conditions that prevented them from completing the questionnaire, who were unable to care for themselves, severely illiterate, and pregnant and lactating women were also excluded. Demographic and medical data were collected.

### Evaluation of DED

All participants completed the OSDI questionnaire, a self-administered questionnaire that assesses the severity of self-reported DED. Based on the total OSDI score, each participant's condition was classified as normal (0–12 points), mild (13–22 points), moderate (23–32 points), and severe (33–100 points). A score of ≥13 points led to a diagnosis of DED.

### Sleep Quality Assessment

Patients' sleep quality in the last 1 month was measured using the Pittsburgh Sleep Quality Index (PSQI). It consists of seven dimensions, namely subjective sleep quality, sleep latency, sleep duration, habitual sleep efficiency, sleep disturbance, use of sleep medicine, and daytime dysfunction. Each dimension is scored on a scale of 0–3, and dimension scores are cumulated to a total PSQI score ranging from 0 to 21, with higher total scores indicating poorer sleep quality. Patients with total scores >7 were considered to have poor sleep.

### Emotion Status Assessment

The Hospital Anxiety and Depression Scale (HADS) questionnaire was created by Zigmond and Snaith (28) to screen for anxiety and depression among patients in general hospitals. The scale contains 14 items, divided into two subscales containing seven items each to assess depression and anxiety. The scale is scored on a 4-point scale (0–3), and the cut-off points for anxiety and depression are total scores of 8 points. HADS has good reliability and validity values (29), and is widely used to screen for anxiety and depression.

### Statistical Analysis

Statistical analyses were performed using the SPSS software (version 23.0; IBM Corp., Armonk, NY, USA).The normality of each continuous variable was tested. Continuous variables that conform to the normal distribution are expressed as Mean ± SD, and those that do not are expressed as median (IQR).Using independent *t*-test or χ2 test for continuous and categorical variables, respectively. When the theoretical frequency (T) was 1 ≤ T < 5, a corrected χ2 test was used.We used Spearson's correlation to analyze the associations between the scores on the OSDI, PSQI and subscales, and HADS. Multiple linear hierarchical regression models were applied to test the mediating role of total PSQI and subscales scores in the relationship of DED with anxiety and depression. Prior to the mediation analysis, all continuous variables were pooled to eliminate multicollinearity. We used *B* as an unstandardized regression coefficient to describe the significant of direct and indirect effects, *Beta* as a standardized regression coefficient to describe the weight of them. The mediating role of sleep quality was further tested using a bootstrap analysis of 5,000 samples. All tests were two-sided, and statistical significance was set at *p* < 0.05.

## Results

This study included 321 patients with DED (89 men and 232 women).The age of them was 48.41 ± 15.15. General patient characteristics are presented in Table 1. We found no difference in sleep between men and women with DED. Menopausal women were more likely to have poor sleep (*p* < 0.05).In addition, age and education levels were also affected factors of sleep (*p* < 0.05).

**Table 1:** Comparison of demographics in patients with DED With good sleep or poor sleep.

**Group**	**DED with good sleep (*n* = 52)**	**DED with poor sleep (*n* = 269)**	**χ^2^**	***P***
**Sex**			0.003	0.959
Male	12 (25.53%)	35 (74.47%)		
Female	40 (20.62%)	154 (79.38%)		
**Menstruation**			6.131	0.013
Menopause	14 (10.85%)	115 (89.15%)		
Non menopause	25 (13.97%)	154 (86.03%)		
**Age^a^**	40.87 ± 14.59	49.87 ± 14.86	4.013^b^	0.000
**Family status**			1.505	0.220
Married	44 (15.33%)	243 (84.67%)		
Single	8 (23.53%)	26 (76.47%)		
**Education levels**				
Primary Education	2 (6.67%)	28 (93.33%)	12.275	0.006
Middle School Education	6 (9.23%)	59 (90.77%)		
High School Education	9 (11.25%)	71 (88.75%)		
University or higher	35 (23.97%)	111 (76.03%)		
**Household location**			0.909	0.340
Urban	44 (17.19%)	212 (82.81%)		
countryside	8 (12.31%)	57 (87.69%)		
**BMI index**			2.370	0.499
<18.5	3 (23.08%)	10 (76.92%)		
18.5 ≤ BMI <25	33 (17.10%)	160 (82.90%)		
25 ≤ BMI <30	12 (12.24%)	86 (87.76%)		
≥30	4 (23.53%)	13 (76.47%)		
**Course of disease**			0.105	0.991
≤ 1 year	24 (15.56%)	124 (84.44%)		
1-3 year	19 (15.70%)	102 (84.30%)		
3-5 year	6 (16.67%)	30 (83.33%)		
≥5 year	3 (18.75%)	13 (81.25%)		
**Frequency of visit (Within one year)**			4.732	0.193
First visit	28 (17.83%)	129 (82.17%)		
<6 times	17 (13.28%)	111 (86.72%)		
6-12 times	3 (12.00%)	22 (88.00%)		
>12 times	4 (36.36%)	7 (63.64%)		

a *Mean ± SD,*

b *independent t-test*.

Among participants, 86 (26.79%), 85 (26.48%), and 54 (16.82%) presented with anxiety, depression, and both anxiety and depression, respectively. A total of 52 (16.20%) patients reported good sleep, and 269 (83.80%) reported poor sleep. In addition, DED patients who had poor sleep were more likely to be anxious and depressed (Table 2).

**Table 2:** Comparison of anxiety and depression in patients with DED with good or poor sleep.

	**DED with good sleep (*n* = 52)**	**DED with poor sleep (*n* = 269)**	***t***	***P***
Anxiety	4.75 ± 3.13	5.90 ± 3.04	−2.475	0.014
Depression	3.96 ± 2.94	5.75 ± 3.20	−3.738	0.000

The mean OSDI score was 45.90 ± 15.90 points. We found that 243 (75.70%) patients with DED were categorized as severe according to the OSDI score, while 18 (5.61%) and 60 (18.69%) patients were categorized as mild and moderate, respectively.

The results of the correlation analyses are presented in Table 3. The OSDI score was significantly correlated with anxiety and depression, as well as with PSQI total score and subjective sleep quality and sleep latency. PSQI total score, subjective sleep quality, and sleep latency were significantly correlated with anxiety and depression.

**Table 3:** Descriptive statistics and intercorrelations between analyzed variables.

	**Descriptive**	**1**	**2**	**3**	**4**	**5**	**6**	**7**	**8**	**9**	**10**	**11**
1. OSDI^a^	45.90 (15.90)	–										
2. Anxiety^a^	5.71 (3.08)	0.189^**^	–									
3. Depression^a^	5.46 (3.22)	0.164^**^	0.643^**^	–								
4. PSQI^a^	14.69 (6.89)	0.181^**^	0.208^**^	0.294^**^	–							
5. SSQ^b^	1.00 (1.00)	0.194^**^	0.168^**^	0.230^**^	0.679^**^	–						
6. SL^b^	1.00 (3.00)	0.122^*^	0.135^*^	0.196^**^	0.611^**^	0.486^**^	–					
7. SD^b^	2.00 (3.00)	0.157^**^	0.014	0.047	0.531^**^	0.466^**^	0.352^**^	–				
8. HSE^b^	1.00 (2.00)	0.186^**^	0.083	0.132^*^	0.574^**^	0.416^**^	0.435^**^	0.630^**^	–			
9. SDE^b^	6.00 (6.00)	0.099	0.196^**^	0.309^**^	0.712^**^	0.285^**^	0.226^**^	0.077	0.193^**^	–		
10. USM^b^	0.00 (0.00)	0.073	0.142^*^	0.170^**^	0.394^**^	0.260^**^	0.248^**^	0.213^**^	0.255^**^	0.113^*^	–	
11. DD^b^	2.00 (2.00)	0.068	0.064	0.061	0.546^**^	0.425^**^	0.282^**^	0.247^**^	0.117^*^	0.123^*^	0.187^**^	–

a *Mean (SD),*

b *Median (IQR),*

* *P < 0.05,*

** *P < 0.01. SSQ, Subjective Sleep Quality; SL, Sleep Latency; SD, Sleep Duration; HSE, Habitual Sleep Efficiency; SDE, Sleep Disturbance; USM, Used Sleep Medication; DD, Daytime Dysfunction*.

Hierarchical regression analyses (Tables 4, 5) revealed that more severe DED (i.e., higher OSDI scores) was associated with poorer subjective sleep quality (B = 0.012, *p* = 0.000), longer sleep latency (B = 0.009, *p* = 0.027), and depressive (B = 0.034, *p* = 0.003) and anxiety symptoms (B = 0.032, *p* = 0.003). Poorer subjective sleep quality (B = 0.411, *p* = 0.024) and longer sleep latency (B = 0.314, *p* = 0.027) were both associated with anxiety symptoms (Table 4). Additionally, poorer subjective sleep quality (B = 0.678, *p* = 0.000) and longer sleep latency (B = 0.447, *p* = 0.002) were both associated with depressive symptoms (Table 5).

**Table 4:** Mediation analysis of PSQI,SSQ,SL on the relationship between the OSDI and anxiety.

	***B***	***Beta***	**SE**	***t***	***P***
OSDI on anxiety	0.032	0.167	0.011	3.030	0.003
OSDI on PSQI	0.085	0.197	0.024	3.581	0.000
PSQI on anxiety (indirect effect)	0.084	0.188	0.025	3.398	0.001
OSDI on anxiety (direct effect)	0.025	0.130	0.011	2.352	0.019
OSDI on anxiety	0.032	0.167	0.011	3.030	0.003
OSDI on SSQ	0.012	0.206	0.003	3.761	0.000
SSQ on anxiety (indirect effect)	0.411	0.127	0.181	2.269	0.024
OSDI on anxiety (direct effect)	0.027	0.141	0.011	2.517	0.012
OSDI on anxiety	0.032	0.167	0.011	3.030	0.003
OSDI on SL	0.009	0.123	0.004	2.222	0.027
SL on anxiety (indirect effect)	0.314	0.123	0.141	2.229	0.027
OSDI on anxiety (direct effect)	0.029	0.152	0.011	2.750	0.006

*SSQ, Subjective Sleep Quality; SL, Sleep Latency*.

**Table 5:** Mediation analysis of PSQI,SSQ,SL on the relationship between the OSDI and depression.

	***B***	***Beta***	**SE**	***t***	***P***
OSDI on depression	0.034	0.168	0.011	3.041	0.003
OSDI on PSQI	0.085	0.197	0.024	3.581	0.000
PSQI on depression (indirect effect)	0.125	0.268	0.025	4.934	0.000
OSDI on Depression (direct effect)	0.023	0.115	0.011	2.119	0.035
				
OSDI on depression	0.034	0.168	0.011	3.041	0.003
OSDI on SSQ	0.012	0.206	0.003	3.761	0.000
SSQ on depression (indirect effect)	0.678	0.201	0.187	3.628	0.000
OSDI on depression (direct effect)	0.026	0.126	0.011	2.284	0.023
				
OSDI on depression	0.034	0.168	0.011	3.041	0.003
OSDI on SL	0.009	0.123	0.004	2.222	0.027
SL on depression (indirect effect)	0.447	0.168	0.147	3.052	0.002
OSDI on depression (direct effect)	0.030	0.147	0.011	2.680	0.008

*SSQ, Subjective Sleep Quality; SL, Sleep Latency*.

**Figure 1:**
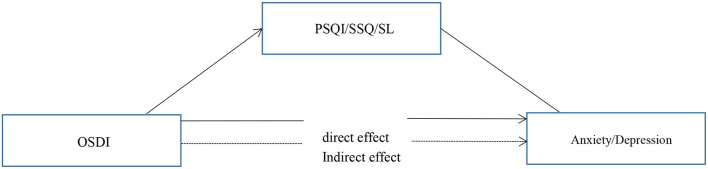
The proposed model of relationships between variables.

Our primary hypothesis (Figure 1) was that the association of DED severity, as measured by the OSDI scores, with depressive and anxiety symptoms would be mediated by poorer subjective sleep quality and longer sleep latency (Table 6). In mediation model 1, the effect of DED on anxiety symptoms was positive and significant, with a standardized estimate of 0.141 (95% CI [0.006–0.048]). This effect was significantly mediated by subjective sleep quality, with a standardized estimate of 0.026 (95% CI [0.001–0.011]).

**Table 6:** Bootstrap results for the mediation analysis.

**Variables**	**Estimate**	**SE**	**LL95%CL**	**UL95%CL**
OSDI on anxiety (direct effect)	0.025	0.011	0.003	0.046
PSQI on anxiety (indirect effect)	0.007	0.037	0.003	0.014
OSDI on depression (direct effect)	0.023	0.115	0.002	0.043
PSQI on depression (indirect effect)	0.011	0.053	0.002	0.013
				
OSDI on anxiety (direct effect)	0.027	0.141	0.006	0.048
SSQ on anxiety (indirect effect)	0.005	0.026	0.001	0.011
OSDI on depression (direct effect)	0.026	0.126	0.005	0.046
SSQ on depression (indirect effect)	0.008	0.041	0.003	0.016
				
OSDI on anxiety (direct effect)	0.029	0.152	0.006	0.050
SL on anxiety (indirect effect)	0.003	0.015	0.001	0.008
OSDI on depression (direct effect)	0.030	0.147	0.008	0.050
SL on depression (indirect effect)	0.004	0.021	0.001	0.010

*SSQ, Subjective Sleep Quality; SL, Sleep Latency*.

In mediation model 2, the effect of DED on anxiety symptoms was positive and significant, with a standardized estimate of 0.152 (95% CI [0.006–0.050]). This effect was significantly mediated by sleep latency, with a standardized estimate of 0.015 (95% CI [0.001–0.008]).

In mediation model 3, the effect of DED on depressive symptoms was positive and significant, with a standardized estimate of 0.126 (95% CI [0.005–0.046]). This effect was significantly mediated by subjective sleep quality, with a standardized estimate of 0.041 (95% CI [0.003–0.016]).

In mediation model 4, the effect of DED on depressive symptoms was positive and significant, with a standardized estimate of 0.147 (95% CI [0.008–0.050]). This effect was significantly mediated by sleep latency, with a standardized estimate of 0.021 (95% CI [0.001–0.010]).

In the four models, the confidence interval excluded zero, indicating a significant indirect effect of DED on anxiety and depressive symptoms *via* mediators.

## Discussion

This study examined the relationship between DED and anxiety and depression, and whether this relationship is mediated by sleep. The results showed that the relationship of DED with anxiety and depression was mediated by subjective sleep quality and sleep latency. Wu et al. (24) found that DED patients with sleep disorders were more likely to be anxious and depressed, which is consistent with our findings.

Eye discomfort symptoms caused by DED may negatively affect mood and mental health (15, 30, 31).The causal relationship between DED and depression remains unclear. However, some factors have been used to explain this association. First, the two diseases are homologous (32); in particular, both share common risk factors, including female sex and menopause. This suggests that sex hormones play an important role in the development of both diseases. Second, somatization is present in 80% of patients with depression (33), which may play a role in exacerbating DED symptoms. In addition, we propose two potential mechanisms: subjective sleep quality and sleep latency, which may help explain the relationship of DED with anxiety and depression.

Several studies (24, 34–37) have found that DED patients suffer from sleep disorders, short sleep duration, poor sleep quality, insomnia, and sleep apnea. This may be due to the fact that tears are produced by the lacrimal glands, which are innervated by the parasympathetic and sympathetic nerves (38). Sleep disorders have been reported to increase the level of stress hormones, including cortisol, epinephrine, and norepinephrine (39), and decrease parasympathetic activity and increase sympathetic tone. Sleep disorders cause mild activation of the hypothalamic-pituitary-adrenal axis in humans, leading to diuresis and excessive natriuresis (40, 41); in addition, the circadian rhythm of hormones in the renin-angiotensin-aldosterone system is significantly altered (41). It has been suggested that a potential mechanism for dehydration in sleep disorder may involve a reduction in nocturnal blood pressure and a decrease in the renin-angiotensin-aldosterone system hormones (42). Altered levels of these hormones and excess diuresis can thus cause a state of relative dehydration, which can affect tear production.

Subjective sleep quality and latency are important components of sleep. Decreased sleep quality disrupts higher-level cognitive functions, such as cognitive control (43, 44). Cognitive control allows for effective emotion regulation (45), and the ability to regulate emotions provides a critical connection between sleep quality and mood disorders (46). Additionally, when participants with sleep problems view negative emotional pictures, the functional connections between brain regions responsible for cognitive control (medial prefrontal areas) and emotional responses (amygdala) are reduced, resulting in poor individual decision-making over time, such as not seeking support, self-harm, persistent fear and distress, and anxiety and depression (47). Cognitive behavioral therapy for insomnia (CBT-I) includes relaxation, stimulus control, and cognitive therapy (48). Moreover, Ashworth et al. (49) found that CBT-I improved subjective and objective sleep quality and reduced depressive symptoms.

Prolonged sleep latency is considered one of the hallmarks of depression (50). Chronic pain and discomfort in DED may cause central sensitization, a common feature of patients with chronic pain; central sensitization is related to the plasticity of the central nervous system (51), a process in which the nervous system's response is progressively enhanced (52), eventually leading to pain despite low levels of peripheral stimulation. This pain response further prolongs sleep latency and ultimately triggers negative mood, anxiety, depressive symptoms in patients, as has been demonstrated in several studies including breast cancer patients (53). During the COVID-19, people were isolated at home and their use of visual display terminal (VDT) devices increased (54).Increased use of VDT can cause many physical discomforts, such as eye strain, musculoskeletal symptoms, headaches, and sleep problem (55). Individuals who use VDT for more than 6 h/day are more likely to have sleep problems (56). Some studies have also found a significant correlation between VDT use and difficulty in falling asleep. Reducing the use of VDT may alleviate the symptoms of DED and increase sleep quality.

This study has several limitations. First, we adopted a cross-sectional study, therefore, it was difficult to establish causal relationships between DED, sleep quality, anxiety, and depression. Second, the current data were collected using self-report scales, with their inherent limitations. Future studies could utilize objective methods, such as polysomnography, to draw more accurate conclusions about sleep outcomes. Third, Galor et al. (36) found that insomnia was more severe in the high pain group of patients with DED. We used the OSDI questionnaire in our assessment of DED, but we did not scale eye pain separately. In future studies we will also focus on eye pain. In addition, the participants in this study were Chinese and constituted an urban community sample from similar areas. Future studies should include larger and more diverse samples.

## Conclusions

This study showed that many patients with DED experience anxiety, depression, and sleep disorders. Our preliminary research supports subjective sleep quality and sleep latency as mediators between DED and both anxiety and depression. These preliminary findings highlight the need to further explore the role of sleep in the relationship between DED and mood.

## Data Availability Statement

The raw data supporting the conclusions of this article will be made available by the authors, without undue reservation.

## Ethics Statement

The studies involving human participants were reviewed and approved by Medical Ethics Committee of Tianjin Medical University Eye Hospital. The patients/participants provided their written informed consent to participate in this study.

## Author Contributions

QH, ZC, and CX: material preparation, data collection, and analysis were performed. The first draft of the manuscript was written by QH and all authors commented on previous versions of the manuscript. All authors contributed to the study conception, design, read, and approved the final manuscript.

## Funding

This work was supported by a grant from the National Natural Science Foundation of China (Grant No: 81770901).

## Conflict of Interest

The authors declare that the research was conducted in the absence of any commercial or financial relationships that could be construed as a potential conflict of interest.

## Publisher's Note

All claims expressed in this article are solely those of the authors and do not necessarily represent those of their affiliated organizations, or those of the publisher, the editors and the reviewers. Any product that may be evaluated in this article, or claim that may be made by its manufacturer, is not guaranteed or endorsed by the publisher.
